# The Activation of the RIG-I/MDA5 Signaling Pathway upon Influenza D Virus Infection Impairs the Pulmonary Proinflammatory Response Triggered by Mycoplasma bovis Superinfection

**DOI:** 10.1128/jvi.01423-22

**Published:** 2023-01-24

**Authors:** Maria Gaudino, Adrien Lion, Eveline Sagné, Brandy Nagamine, Justine Oliva, Olivier Terrier, Elisabeth Errazuriz-Cerda, Anaëlle Scribe, Fatima-Zohra Sikht, Elisa Simon, Charlotte Foret-Lucas, Blandine Gausserès, Julie Lion, Ana Moreno, Emilie Dordet-Frisoni, Eric Baranowski, Romain Volmer, Mariette F. Ducatez, Gilles Meyer

**Affiliations:** a IHAP, Université de Toulouse, INRAE, ENVT, Toulouse, France; b Centre International de Recherche en Infectiologie – U1111 (Equipe VirPath) – Institut National de la Santé et de la Recherche Médicale, Ecole Normale Supérieure, Lyon, France; c Centre National de la Recherche Scientifique – UMR5308, Lyon, France; d Centre d’Imagerie Quantitative Lyon 1, Lyon, France; e Istituto Zooprofilattico Sperimentale della Lombardia e dell’Emilia Romagna “Bruno Ubertini,” Brescia, Italy; Emory University School of Medicine

**Keywords:** coinfections, mycoplasma, precision-cut lung slices, PCLS, cattle, *ex vivo*, influenza, respiratory viruses

## Abstract

Concurrent infections with multiple pathogens are often described in cattle with respiratory illness. However, how the host-pathogen interactions influence the clinical outcome has been only partially explored in this species. Influenza D virus (IDV) was discovered in 2011. Since then, IDV has been detected worldwide in different hosts. A significant association between IDV and bacterial pathogens in sick cattle was shown in epidemiological studies, especially with Mycoplasma bovis. In an experimental challenge, IDV aggravated M. bovis-induced pneumonia. However, the mechanisms through which IDV drives an increased susceptibility to bacterial superinfections remain unknown. Here, we used the organotypic lung model precision-cut lung slices to study the interplay between IDV and M. bovis coinfection. Our results show that a primary IDV infection promotes M. bovis superinfection by increasing the bacterial replication and the ultrastructural damages in lung pneumocytes. In our model, IDV impaired the innate immune response triggered by M. bovis by decreasing the expression of several proinflammatory cytokines and chemokines that are important for immune cell recruitment and the bacterial clearance. Stimulations with agonists of cytosolic helicases and Toll-like receptors (TLRs) revealed that a primary activation of RIG-I/MDA5 desensitizes the TLR2 activation, similar to what was observed with IDV infection. The cross talk between these two pattern recognition receptors leads to a nonadditive response, which alters the TLR2-mediated cascade that controls the bacterial infection. These results highlight innate immune mechanisms that were not described for cattle so far and improve our understanding of the bovine host-microbe interactions and IDV pathogenesis.

**IMPORTANCE** Since the spread of the respiratory influenza D virus (IDV) infection to the cattle population, the question about the impact of this virus on bovine respiratory disease (BRD) remains still unanswered. Animals affected by BRD are often coinfected with multiple pathogens, especially viruses and bacteria. In particular, viruses are suspected to enhance secondary bacterial superinfections. Here, we use an *ex vivo* model of lung tissue to study the effects of IDV infection on bacterial superinfections. Our results show that IDV increases the susceptibility to the respiratory pathogen Mycoplasma bovis. In particular, IDV seems to activate immune pathways that inhibit the innate immune response against the bacteria. This may allow M. bovis to increase its proliferation and to delay its clearance from lung tissue. These results suggest that IDV could have a negative impact on the respiratory pathology of cattle.

## INTRODUCTION

Mixed airways infections, with pathogenic bacteria and viruses often codetected in respiratory secretions, are common in humans and animals (1). Many of these microbial associations are now considered to negatively affect the clinical outcome of infection (2–4). Cattle are no exception, and respiratory outbreaks in this species have a multifactorial origin that often involves several pathogens (5–8). Coinfections are frequently associated with an increase in severity of disease and, in some cases, a decrease in the survival rate (9–14). While data illustrating the clinical importance of mixed respiratory tract infections are accumulating, the mechanisms associated with coinfections are still poorly understood (15). New respiratory viruses have been recently discovered in cattle. These include the influenza D virus (IDV), a novel *Orthomyxoviridae* isolated in 2011 from swine with influenza-like illness (16). IDV exposure was documented in humans by serologic studies (17–19) and in several animal species (20–24), but cattle are considered its primary host (25). In the last few years, IDV detection in clinical samples has been positively associated with respiratory disease in cattle through metagenomic approaches (26, 27). In addition, epidemiological studies showed a significant association between IDV and Mycoplasma bovis in sick cattle (5). Experimental *in vivo* challenge with IDV revealed viral replication in both the upper and lower respiratory tracts with moderate respiratory sign onset in calves (28, 29) and subacute bronchointerstitial pneumonia with neutrophil infiltration in bronchial lumens and neutrophilic and macrophagic alveolitis at necropsy (29). Although the clinical signs induced by IDV infection were moderate, in experimental coinfection, IDV enhanced M. bovis colonization of the lower respiratory tract leading to exacerbated clinical manifestations (30). The mechanisms underlying the increase in susceptibility to bacterial superinfections following IDV infection remain, however, unknown.

The present study aimed to decipher the mechanisms underlying IDV and M. bovis synergistic interplay upon airway colonization by using precision-cut lung slices (PCLSs) as an organotypic lung model of infection. PCLS represents a versatile tool that has the huge benefit of meeting the 3Rs principle (replacement, reduction, and refinement) to reduce the number of animals used in *in vivo* experiments. It has been extensively used for lung disease modeling (31–33), including studies with bovine pathogens (34–36). Additional advantages of this model include the preservation of the three-dimensional structure and physiological properties of the lung tissue, the preservation of the resident cells that were present in the collected organs, and the possibility of doing a large number of biological replicates for different experimental conditions (37). We studied the respiratory tropism of each pathogen, their replication, the cellular ultrastructure modifications, and the inflammatory and innate immune responses in monoinfections or when a primary IDV infection was followed by M. bovis superinfection.

## RESULTS

### Viability of organotypic PCLS cultures.

Bovine PCLS cultures were used to study the dynamics of IDV and M. bovis respiratory infections *ex vivo*. Before investigating the impact of mixed infections, we first established the viability of PCLS cultures over 7 days postslicing by two different methods. We stained PCLS single-cell suspensions with 7-aminoactinomycin D (7-AAD), and we measured 7-AAD-stained cells by flow cytometry. In parallel, we measured the cytotoxicity by quantifying the lactate dehydrogenase (LDH) activity in PCLS supernatants.

**FIG 1:**
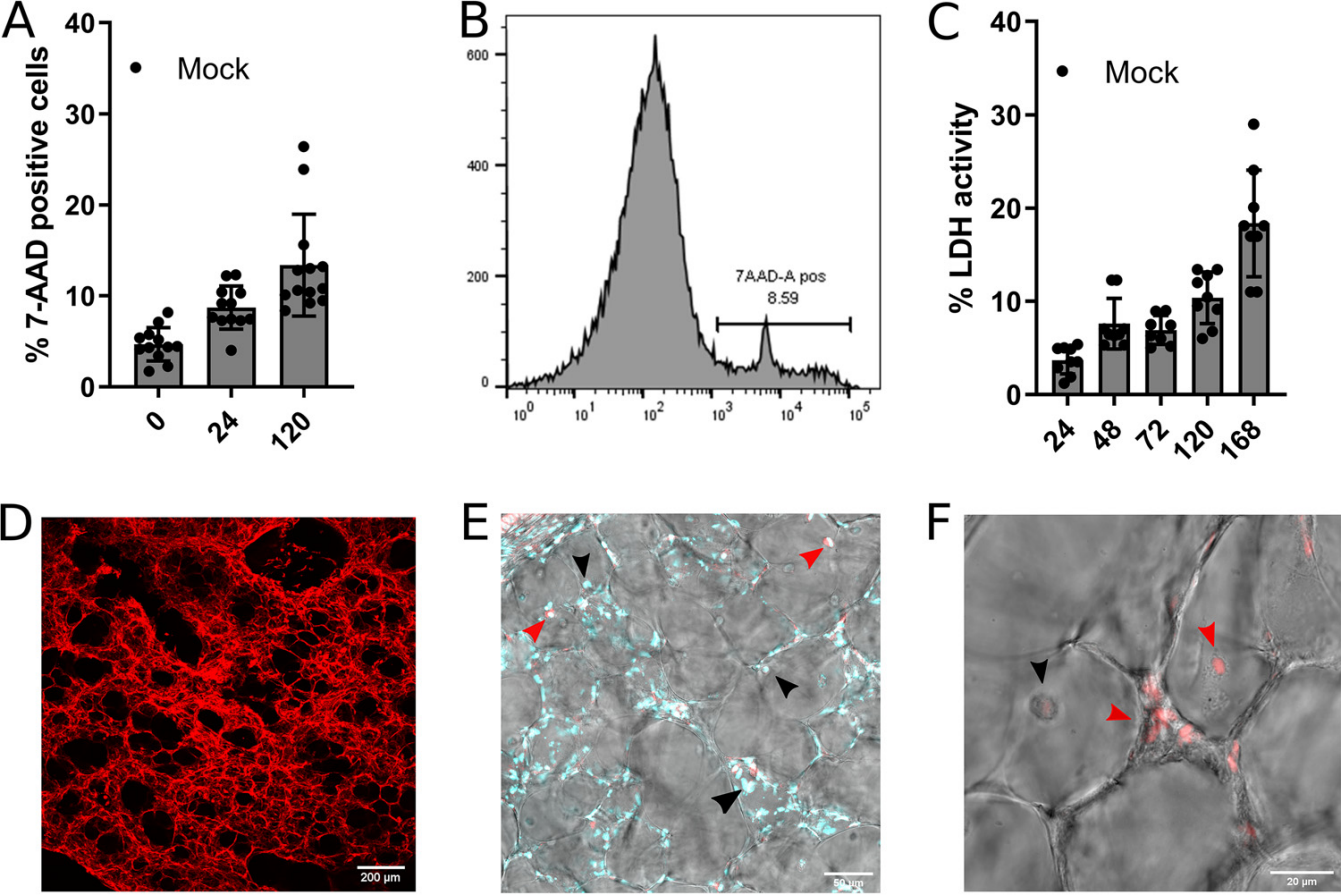
Viability of organotypic precision-cut lung slice (PCLS) cultures. (A) Percentage of 7-aminoactinomycin D (7-AAD)-positive cells in noninfected PCLSs at different time points (h). The measures were performed in triplicate on four different lung donors in four independent experiments (each dot represents the measured value for a single lung slice). The results are expressed as means ± standard error of the mean (SEM). (B) Example of flow cytometry profile of PCLS apoptotic cells stained with 7-AAD. The *x* axis of the histogram graph represents the relative fluorescence, and the *y* axis represents the number of events. (C) Percentage of lactate dehydrogenase (LDH) activity in supernatants of noninfected PCLSs at different time points (h). The measures were performed in triplicate on four different lung donors in four independent experiments. Each dot represents the measured value for a single lung slice. The results are expressed as means ± SEM. (D) β-Tubulin staining (red) of mock PCLSs. (E) Viable cells (indicated with the black arrowheads) and necrotic cells on the inner part of PCLSs positive for propidium iodide stain (indicated with the red arrowheads). The cell nuclei in cyan were stained with 4′,6-diamidino-2-phenylindole (DAPI). (F) Necrotic cells on the apical surface of PCLSs positive for propidium iodide stain (indicated with red arrowheads).

Flow cytometry analysis of 7-AAD-positive cells revealed a low percentage of apoptotic cells at 24 h postslicing (<12%), with higher values at 120 h (11% to 30%) (Fig. 1A and B). The LDH activity remained low up to 120 h postslicing (<15%), with a decline in viability detectable only at day 7 postslicing (10% to 30% of LDH activity) (Fig. 1C). β-Tubulin staining indicated a well preserved alveolar structure of cultured PCLSs (Fig. 1D). To further characterize the viability of mock-infected PCLS cultures, necrotic cells were visualized by propidium iodide (PI) staining and confocal imaging on multiple confocal plans at 72 h postslicing. PI-stained cells, mainly pneumocytes and resident alveolar macrophages, were only observed on apical and basal plans (Fig. 1F), whereas cells in the inner part of the slices remained viable (Fig. 1E). Finally, the ciliary activity was daily observed under a light microscope. All mock-infected PCLSs displayed ciliary motion in lung airways until the end of the experiment (7 days postslicing). Overall, these data suggest that PCLS cultures showed good cell viability for up to 5 days, with an increase in cell mortality on day 7. As a consequence, for coinfection experiments, PCLSs were cultured until a maximum of 5 days postslicing.

### Bovine PCLSs are permissive to IDV and M. bovis infection and reproduce *in vivo*-like innate immune response.

**FIG 2:**
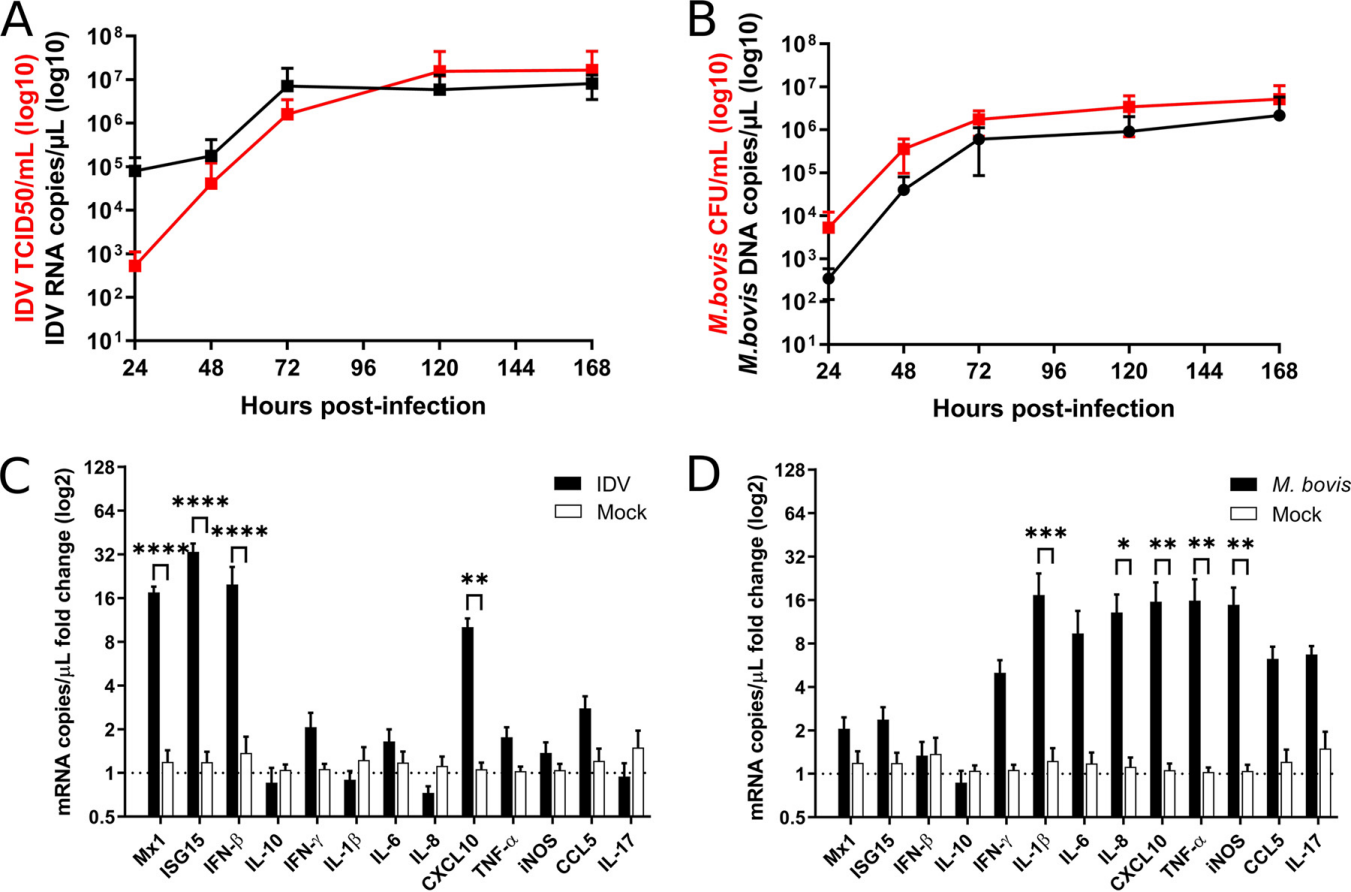
Replication kinetics and immune response of influenza D virus (IDV) and M. bovis on PCLSs. (A, B) Replication of IDV by viral titration and reverse transcription quantitative PCR (RT-qPCR) (A) and of M. bovis by bacterial enumeration and qPCR at different time points (B). For replication kinetics study, the infections were done with a multiplicity of infection (MOI) of 0.001 (10³ 50% tissue culture infective dose [TCID_50_]/PCLS for IDV, 10³ CFU/PCLS for M. bovis). The measures were performed in triplicate on three different lung donors in three independent experiments. The results are expressed as means ± SEM. (C, D) RT-qPCR profiles of different cytokines induced on PCLSs following IDV (C) and M. bovis (D) infection on PCLSs at 48 h p.i. For the innate immune response profiling, the infections were done with an MOI of 1 (10^6^ TCID_50_/PCLS for IDV, 10^6^ CFU/PCLS for M. bovis). The measures were performed in triplicate on three different lung donors in three independent experiments. The results are expressed as means ± SEM. IFN, interferon; IL, interleukin; iNOS, inducible nitric-oxide synthase; TNF, tumor necrosis factor.

We then established the permissiveness of this model for each pathogen by studying their replication and the innate immune response. The replication of individual pathogens in PCLS cultures was determined by both titration and reverse transcription quantitative PCR (RT-qPCR) for IDV and bacterial numeration and qPCR for M. bovis (Fig. 2A and B). IDV and M. bovis displayed a 4-log_10_ increase in replication from 24 to 168 h post-IDV infection (p.i.), indicating that the PCLS model is permissive for both pathogens. Interestingly, infected PCLSs failed to reveal enhanced cytotoxicity compared to mock conditions up to 7 days p.i. (8 days postslicing) (Fig. S1). The PCLS innate immune response to individual infections was then assessed by RT-qPCR for a panel of cytokines at 48 h p.i. Representative cytokines for the innate immune pathways activated upon IDV and M. bovis infection were selected based on transcriptomic data that were previously obtained *in vivo* (29, 30).

The innate immune response to IDV infection was characterized by the activation of type I interferon (IFN-β), Mx1, and ISG15 (Fig. 2C), as well as CXCL10. In contrast to IDV, M. bovis induced an upregulation of proinflammatory cytokines, especially interleukin-1β (IL-1β), IL-8, CXCL10, and tumor necrosis factor α (TNF-α) (Fig. 2D), together with the inducible nitric-oxide synthase (iNOS), the expression of which was associated with pneumonic lesions *in vivo* (38). In addition, IL-10 response was not observed. Overall, these data suggest that PCLS cultures allow the replication of both pathogens and the initiation of an innate immune response similar to those observed *in vivo* (29, 30).

### PCLS infections with IDV and M. bovis revealed a similar distribution and tissue tropism.

**FIG 3:**
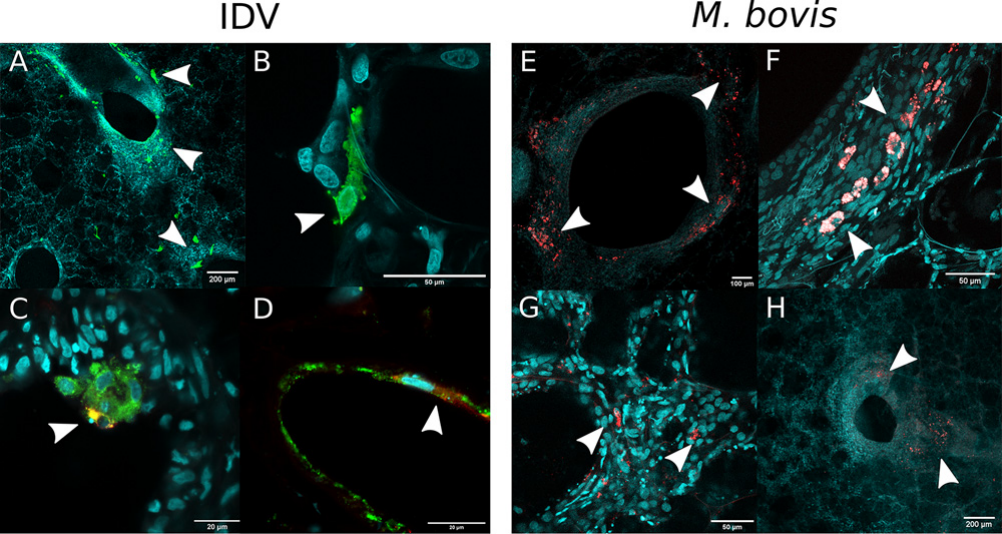
Confocal microscopy studies of IDV and M. bovis distribution on PCLSs infected with an MOI of 0.001 (10³ TCID_50_/PCLS for IDV, 10³ CFU/PCLS for M. bovis). In all the images, the nuclei were stained with DAPI (cyan). (A) Z-stack image of IDV (green) infecting alveolar parenchyma and bronchial cells at 48 h p.i. (B) An alveolar pneumocyte infected by IDV at 48 h p.i. (C) IDV (red) infecting endothelial cells (green) at 48 h post-IDV infection (p.i.). (D) IDV (red) infecting Club cells (green) in bronchioles at 48 h p.i. (E) M. bovis mCherry (red) infecting bronchiolar cells at 120 h p.i. (F) Z-stack image of M. bovis mCherry (red) infecting bronchiolar cells at 120 h p.i. (G, H) M. bovis mCherry (red) infecting the alveolar parenchyma at 120 h p.i. (G) and bronchiolar cells (H).

**FIG 4:**
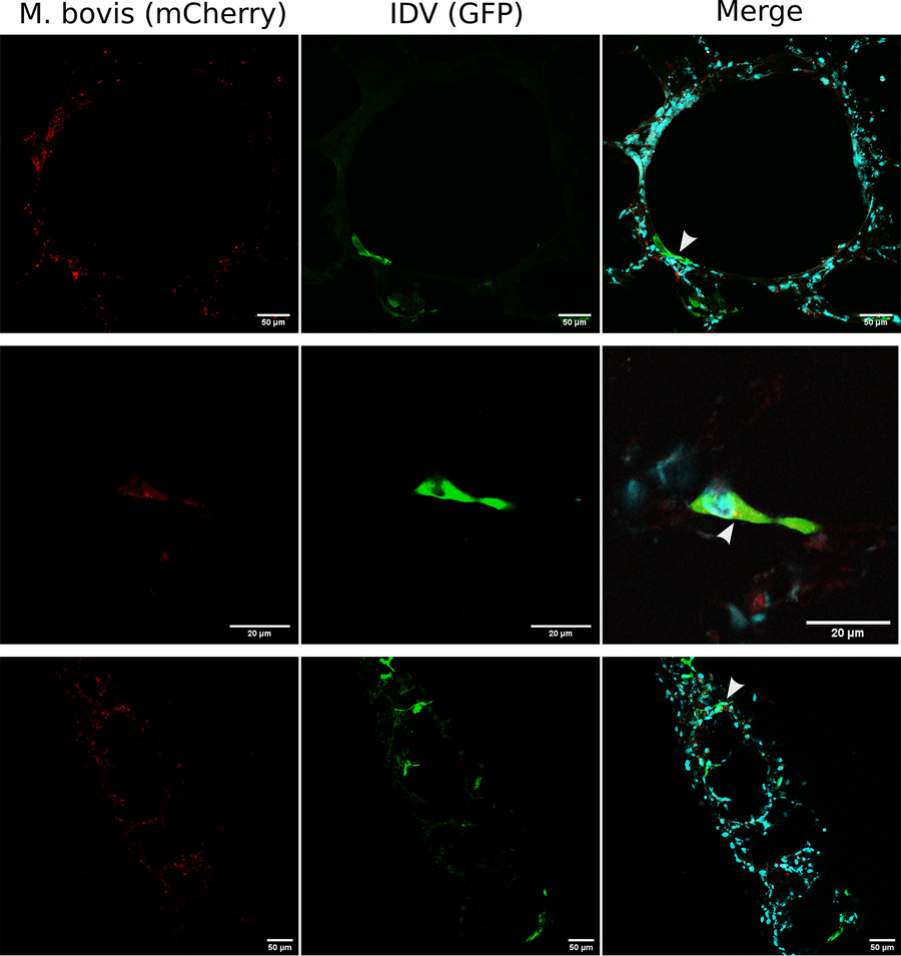
Confocal microscopy studies of M. bovis (red) and IDV (green) colocalization on PCLSs at 48 and 72 h p.i. The PCLSs were infected with an MOI of 0.001 (10³ TCID_50_/PCLS for IDV, 10³ CFU/PCLS for M. bovis). The nuclei were stained with DAPI (cyan). The white arrows on merged images indicate the colocalization of the two pathogens.

Immunofluorescence with confocal microscopy imaging was then carried out on PCLSs to study the distribution and the tissue tropism of each pathogen. Fig. S3 shows confocal images taken on mock PCLSs. Z-stack imaging revealed that IDV infects predominantly bronchial epithelial cells but also the alveolar parenchyma (Fig. 3A and B) and Club cells (Fig. 3D), which are epithelial bronchiolar exocrine cells secreting glycosaminoglycans that protect the bronchiolar epithelium (39). Less frequently, IDV was also localized in MHC-II expressing cells (Fig. S2E and F) and endothelial cells (Fig. 3C), the latter cell type being a hallmark of pathogenic influenza A virus infections (40). For M. bovis, fluorescent foci of bacterial replication and colonies were observed particularly in bronchioles (Fig. 3E, F, and H) and Club cells (Fig. S2G) but also in the alveolar parenchyma (Fig. 3G), similar to what was observed for IDV. By confocal microscopy, we could also colocalize IDV and M. bovis in coinfected conditions, as shown in Fig. 4. IDV and M. bovis infected similar cell types, namely, bronchial cells (Fig. 4, top panel) and alveolar pneumocyte (Fig. 4, middle and bottom panels), strengthening the notion of a similar tissue tropism.

### IDV promotes M. bovis replication and increases the ultrastructural changes in bovine PCLSs.

**FIG 5:**
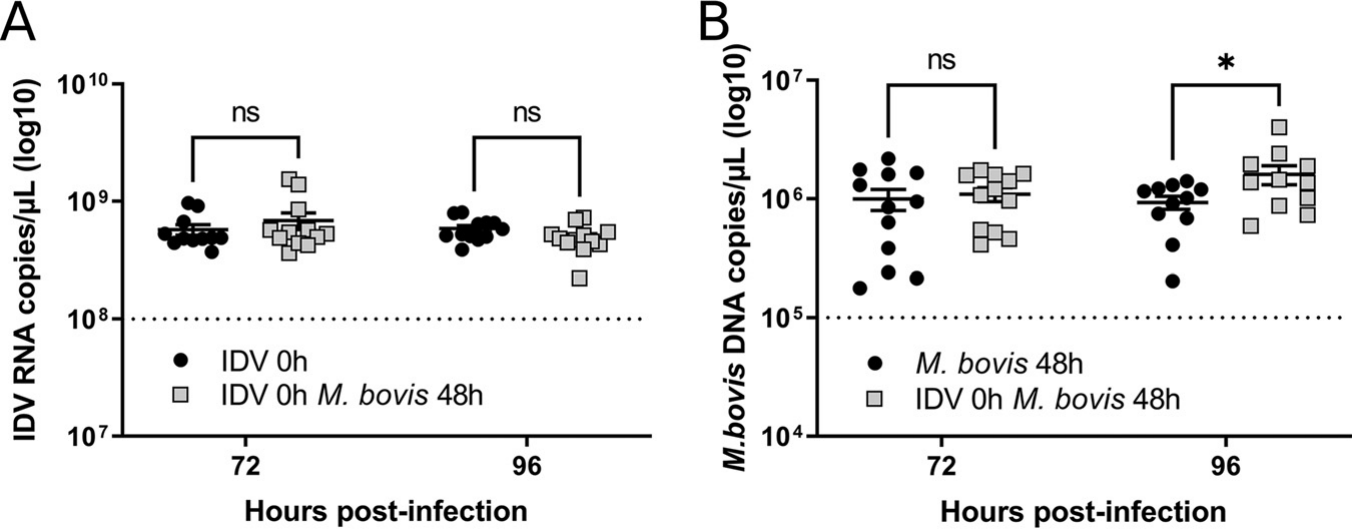
Genomic copies of IDV (A) and M. bovis (B) measured by (RT)-qPCR in supernatants of superinfected conditions. The dotted line represents the number of copies in the inoculum of each pathogen. The PCLSs were infected with an MOI of 1 (10^6^ TCID_50_/PCLS for IDV, 10^6^ CFU/PCLS for M. bovis). The measures were performed in triplicate on four different lung donors in three independent experiments. The results are expressed as means ± SEM. ns, not significant.

Our results indicate that our PCLS model is permissive for IDV and M. bovis in monoinfection, and it reproduces *in vivo*-like innate immune response upon infection. In addition, a similar tissue tropism for both pathogens was highlighted by confocal microscopy studies. We then investigated the interactions between the two pathogens in coinfected conditions compared to monoinfections. In particular, to study the effects of a primary IDV infection on M. bovis superinfection, the PCLSs were infected at 0 h with IDV and 48 h later with M. bovis. The supernatants and lung cuts were then collected at 72 and 96 h p.i. While no changes in IDV replication were observed between single and superinfected conditions (Fig. 5A), we observed that preinfection with IDV caused an approximately 2-fold increase in M. bovis replication at 96 h p.i. (Fig. 5B).

**FIG 6:**
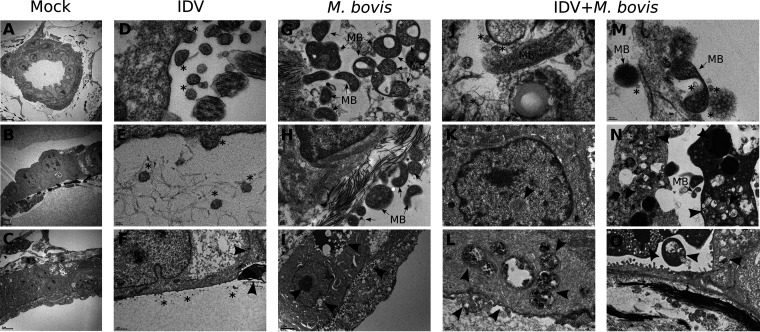
Transmission electronic microscopy of single IDV and M. bovis or coinfection on PCLSs. All images presented here were taken at 48 and 72 h p.i. The PCLSs were infected with an MOI of 1 (10^6^ TCID_50_/PCLS for IDV, 10^6^ CFU/PCLS for M. bovis). The black arrows indicate the cell ultrastructural changes. *, IDV; MB, M. bovis. (A to C) Noninfected PCLSs (mock) presented complex structure with polarized epithelial cells associated with red and white blood cells. (D to F) Single IDV infection induced damages on ciliated epithelial cell, with loss of cilia, cell disorganization, and inflammation. (G) M. bovis in active division. (H, I) M. bovis infection caused cell apoptosis and mucus production with bacteria aggregate on the apical side. (J, M) IDV and M. bovis colocalization; the viral particles and the bacteria can be seen in close contact with each other. (K) Intranuclear inclusions observed upon coinfection. (L) Viro-induced structures upon coinfection. (N, O) Cell death upon IDV and M. bovis infection.

Ultrastructural changes associated with single or coinfections were analyzed by transmission electron microscopy. Single infections with each pathogen (Fig. 6D and I) affected the structure of the respiratory epithelium compared to mock conditions (Fig. 6A to C). Localized on the apical side of the epithelium, IDV was mainly associated with ciliated cells and pneumocytes inducing a loss of cilia, nuclear inclusions (Fig. 6F), as well as cell disorganization likely caused by a loss of tight junctions (Fig. 6F). Similarly, M. bovis was also localized on the apical side of the epithelium and remained mainly extracellular (Fig. 6H). M. bovis induced more ultrastructural changes in lung cells than IDV. Damages associated with M. bovis infection included dense ultrastructure in the cytoplasm (Fig. 6I), cell death (apoptosis and autophagy), and mucus production (Fig. 6I).

Upon coinfections, we confirmed IDV and M. bovis colocalization in alveolar parenchyma, suggesting a close contact between the two pathogens (Fig. 6J and M). The coinfection presented damages with a composite pattern of both single infections. Coinfected PCLSs presented nuclear inclusions, as shown in Fig. 6K. Similar signs of both single infections were observed but with higher intensity compared to monoinfections, characterized by an increase in viro-induced ultrastructures (Fig. 6L), as well as phagophores (Fig. S4), which are correlated with autophagy. An increase in cell death was observed in coinfected conditions compared to monoinfections (Fig. 6N and O). Despite this, it is not known whether the increased cell death can be attributed to IDV or M. bovis upon coinfection.

### IDV infection impairs the proinflammatory responses triggered by M. bovis superinfection.

**FIG 7:**
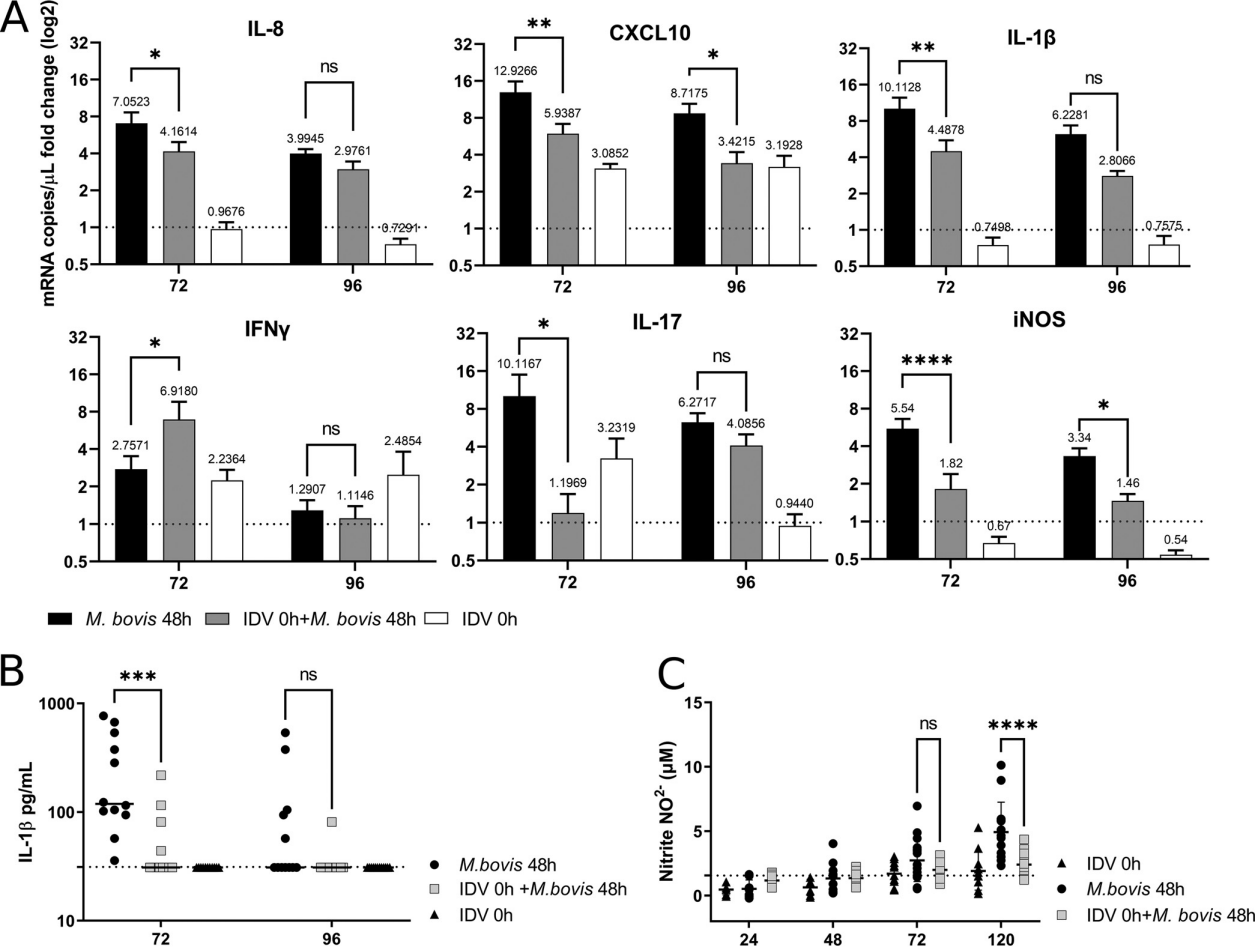
Impact of IDV on the proinflammatory and antibacterial immune response against M. bovis at different time points. (A) RT-qPCR analysis of the expression of different cytokines measured by RT-qPCR in infected lung tissue. The dotted line represents the fold change of mock PCLSs. The PCLSs were infected with an MOI of 1 (10^6^ TCID_50_/PCLS for IDV, 10^6^ CFU/PCLS for M. bovis). The plotted values above bars represent the mean for each group. (B) IL-1β protein quantification in infected supernatants by ELISA. The dotted line represents the limit of detection of the commercial kit. (C) Nitrite quantification in infected supernatants by Griess assay. The dotted line represents the mean values of mock conditions. For panels A to C, the measures were performed in triplicate on four different lung donors in three independent experiments. The results are expressed as means ± SEM.

We then investigated the impact of coinfection on the innate immune response compared to monoinfections. The RT-qPCR analysis revealed that the proinflammatory response against M. bovis was decreased in the coinfected conditions (Fig. 7A). The mRNA expression of several cytokines induced by M. bovis, including IL-8, IL-1β, and IL-17, was significantly decreased at 72 h p.i. in coinfected conditions but recovered their baseline levels of expression 1 day later, whereas CXCL10 and iNOS remained statistically decreased also at 96 h p.i. No effect was observed on Mx1 response (Fig. S5, supplemental material) in coinfected conditions, which is probably linked to no changes in IDV replication (Fig. 5A). No differences were observed for IL-6 and CCL5 between mono- and coinfected conditions (Fig. S5, supplemental material). Interestingly, the only upregulated cytokine in coinfected conditions was IFN-γ, similar to what we previously observed *in vivo* (30). The decreased IL-1β production at 72 h p.i. (Fig. 7B) and iNOS lower activity (Fig. 7C) were confirmed by enzyme-linked immunosorbent assay (ELISA) assay and Griess assay, respectively.

### The NF-κB pathway contributes to the control of M. bovis replication.

**FIG 8:**
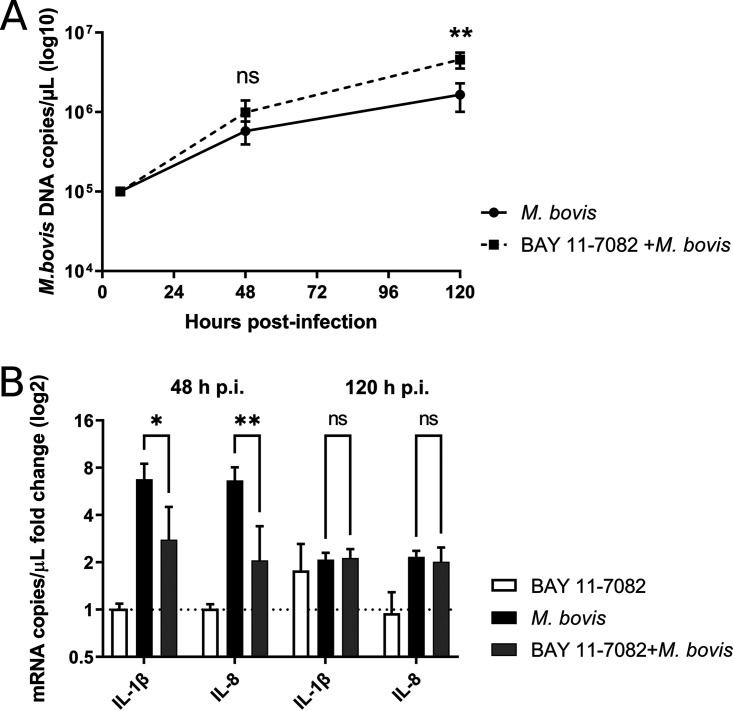
Impact of the inhibition of NF-κB signaling pathway on M. bovis (10^6^ CFU/mL). (A, B) replication (A) and proinflammatory response (B) at 48 and 120 h p.i. The dotted line represents the fold change of mock PCLSs. The measures were performed in triplicate on two different lung donors. The results are expressed as means ± SEM.

Activation of the NF-κB pathway has been described for Mycoplasma spp. infection in different species (41, 42), including bovine (43), via Toll-like receptor 2 (TLR2) binding and MyD88-dependent signaling. Our RT-qPCR data on cytokine transcripts (Fig. 7A) suggest that IDV may counteract this pathway, leading to decreased expression of several cytokines and chemokines and increased bacterial replication. To confirm the role of proinflammatory cytokines in the control of M. bovis infection, we pretreated the PCLSs with an inhibitor of NF-κB (BAY 11-7082) for 6 h, followed by the inoculation of M. bovis on treated and untreated lung slices. The decreased expression of IL-1β and IL-8 mRNAs at 48 h p.i. was associated with an increase in M. bovis replication in both lung donors at 120 h p.i. (Fig. 8), similar to what has been observed in superinfection (Fig. 5B). This suggests that the NF-κB pathway plays a role in the control of M. bovis replication in bovine lung.

### RIG-I/MDA5 activation desensitizes the TRL2 signaling pathway.

**FIG 9:**
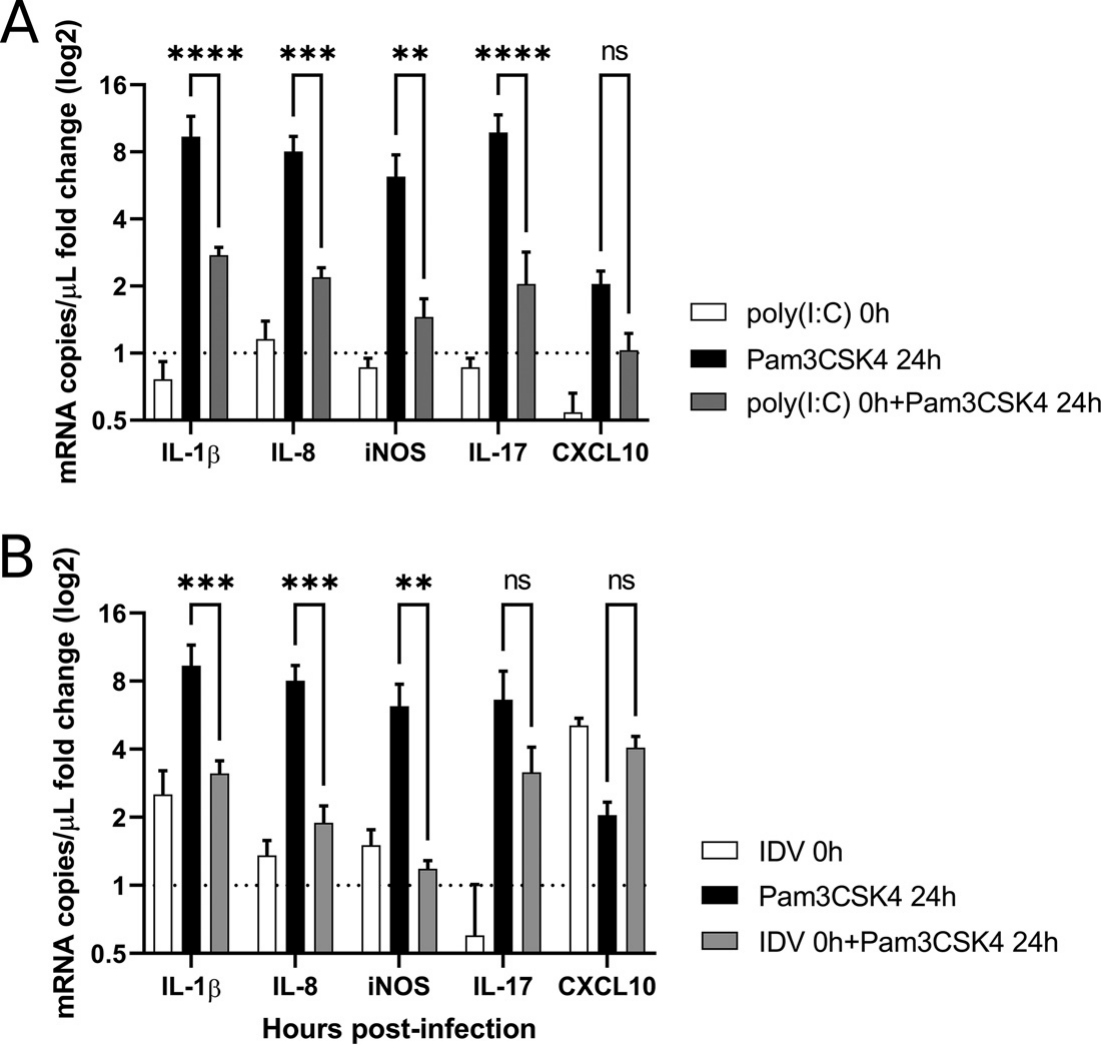
Effects of the activation of the RIG-I/MDA5 signaling pathway on the proinflammatory cytokines activated via the TLR2 pathway at 72 h p.i. RIG-I/MDA5 was activated by poly(I·C) LyoVec stimulation (A) and by IDV infection (B) (10^6^ TCID_50_/mL). The dotted line represents the fold change of mock PCLSs. The measures were performed in triplicate on four different lung donors in three independent experiments. The results are expressed as means ± SEM.

To investigate the mechanisms that drive a decreased expression of proinflammatory cytokines in the coinfected conditions, we used synthetic agonists that activate specific immune pathways. In particular, IDV is known to stimulate the RIG-I/MDA-5 cytosolic receptor, whereas M. bovis is known to activate the TLR2 membrane receptor upon infection (29, 43–45). As shown in Fig. 9A, a primary activation of RIG-I/MDA5 induced by intracellularly delivered poly(I·C) stimulation desensitizes the TLR2 pathway cytokines induced by Pam3CSK4 agonist added 24 h later, suggesting a cross talk between cytosolic helicases and TLRs. Similar results were also obtained when IDV infection was used instead of poly(I·C) stimulation (Fig. 9B) with significant decreases of IL-1β, IL-8, and iNOS mRNAs loads. IL-17 mRNA levels also decreased in the presence of poly(I·C) pretreatment; however, the differences were not significant.

## DISCUSSION

An organotypic PCLS model was used in this study to better characterize IDV and M. bovis infections in lung and to decipher the molecular mechanisms explaining the impact of a primary IDV infection on M. bovis superinfection. In the present study, we focused on a primary viral infection, as in the literature it was described to be one of the most common triggers for bovine respiratory disease in the field (38). The use of this model could be useful in the future to investigate further conditions of coinfections. Both pathogens had a particular tropism for bronchiolar epithelial cells, which is consistent with the respiratory signs observed in infected animals (29, 30). The colocalization of IDV and M. bovis in mixed infections points toward a similar tropism. A possible aggregation between these two pathogens has been suggested by several electron microscopy images, but the biological significance of these particular structures remains to be further investigated. In particular, the impact of these structures on the transmission of both pathogens simultaneously among animals could be studied in more depth.

Our RT-qPCR data revealed a downregulation of mRNA expression of proinflammatory cytokines upon IDV infection, which were confirmed by ELISA for IL-1β and Griess test for iNOS, suggesting that this virus could negatively regulate the NF-κB pathway in the first days of infection, a mechanism that is commonly exploited by several viruses to evade the immune response (46). The impairment of proinflammatory cytokines and chemokines that play a key role in neutrophil recruitment may influence the response against M. bovis infection, thereby promoting mycoplasma growth, as we experimentally confirmed by inhibiting the NF-κB pathway. This is in agreement with what was observed for the human pathogen Mycoplasma pneumoniae in a mouse model in which mice unable to produce IL-1β had delayed bacterial clearance in the lungs (47). Attenuated IL-1β production was also linked with Staphylococcus aureus pneumonia exacerbation in another study (48). Previously described pathogenic mechanisms of human influenza A virus (IAV) in a mouse model include neutrophil impairment in lung associated with increased susceptibility to Streptococcus pneumoniae (49) and neutrophil chemoattractant deficiency that resulted in the inability to efficiently resolve S. pneumoniae superinfection (50). In other studies, experimental pneumococcal superinfections aggravation were associated with IFN-I presence, as shown by the increased survival of Ifnar^−/−^ mice compared to wild-type mice (51, 52) and to decreased Th17 response (53), as shown by the impaired S. aureus clearance in IL-17R^−/−^ mice compared to wild-type mice. Similarly, in our study, we observed that IDV decreased IL-17 mRNA of 10-fold in superinfection. In our model, we could confirm the decreased proinflammatory and chemokines expression by RT-qPCR and ELISAs; however, the immune cell recruitment in lung, which plays a very important role in the outcome of the coinfection, cannot be studied.

Interestingly, IFN-γ was the only upregulated cytokine in this study, similar to what was observed during *in vivo* trial with IDV and M. bovis, where IFN-γ upregulation was linked to increased lung lesions in coinfected animals (30). This confirms previous studies that described the role of IFN-γ on alveolar macrophage depletion and a consequent delayed bacterial clearance (54). In addition, IFN-γ signaling was also shown to impair cell recruitment during the progression of influenza/S. pneumoniae coinfection (55).

The role of IDV in predisposing organisms to secondary bacterial superinfections was previously investigated *in vivo*. Mice were first inoculated with IDV, and 7 days later they were challenged with S. aureus. However, IDV antiviral response had a protective effect on coinfected animals by increasing the survival rate and recovery compared to the S. aureus group alone (56), contrary to what was reported for IAV primary infection and S. aureus superinfection in the same model, in which IAV primed and predisposed mice to secondary pneumonia (57, 58). IAV-solicited host immune factors are similar to those observed in IDV infection, with an induction of interferon-stimulated genes (ISGs) and antiviral proteins. Concurrent with their antiviral effect, type I IFN production can decrease important antibacterial immune responses and neutrophil-recruiting chemokines (58), which is a mechanism similar to what we observed with IDV and M. bovis coinfection in this study. Therefore, the outcome of coinfections seems to be host- and pathogen-dependent, and future studies should focus on the interspecies variability to IDV immune response in coinfection.

To investigate the mechanisms that drive a decreased expression of proinflammatory cytokines and chemokines in coinfection in our model, we used synthetic agonists that activate specific immune pathways that mimic viral and mycoplasma infections. Our results suggest that interference between cytosolic RIG-I-like receptors (RLRs) and membrane TLR2 receptor activation takes place. In our *ex vivo* conditions, the observed downregulation of proinflammatory cytokines was, however, transient, and our observations in time were limited at 72 h poststimulation with pattern recognition receptor (PRR) agonists and at 96 h p.i. with the replicating pathogens. The interference between TLR2 and RIG-I/MDA5 is in agreement with the results observed in bone marrow-derived macrophages in mice (59). Similarly, the activation of MyD88, which is pivotal for the signaling of membrane Toll-like receptors, was observed to be a negative regulator for the TLR3/TRIF pathway in mice corneal epithelium (60). In addition, MyD88 activation was also shown to inhibit TRIF-mediated IFN-β and RANTES by suppressing IκB kinase ε (IKKε)-dependent IRF3 phosphorylation in macrophages (61). The interactions among different PRRs could range from nonadditive to desensitizing responses, which may have a negative impact when coinfecting pathogens that activate different innate pathways are present (62). Altogether, these results suggest that the interference between different PRRs represents a possible mechanism for the increased susceptibility to respiratory disease in viral and bacterial coinfections, at least for IDV and M. bovis. We observed that the interference between RIGI/MDA5 and TLR2 was stronger with poly(I·C)/LyoVec and Pam3CSK4 treatments than with coinfections with the two pathogens. This could be due to the activation of additional PRRs that have not been described for IDV and M. bovis so far.

IDV matrix protein was shown to suppress RLR and NF-κB signaling in human cells HEK-293T by degrading the TNF receptor-associated factor 6 (TRAF6) (63), which is known to play a pivotal role in NF-κB activation (64–66) but also in the IFN-I pathway (67). Our RT-qPCR and ELISA results in bovine PCLSs indicate that a counteraction of the NF-κB pathway takes place also in cattle. In addition to RIG-I/MDA5 activation, the degradation of TRAF6 upon primary IDV infection could be a mechanism that explains the downregulation of the TRL2-induced cytokines via the TRAF6/NF-κB pathway.

In our previous *in vivo* trial, animals coinfected with IDV and M. bovis had an increased bacterial colonization in the lower respiratory tract, which was linked to an increase in the clinical scores and gross lung lesions compared to the group challenged with M. bovis alone (30). Taken together, our *in vivo* and *ex vivo* results suggest that IDV could have an impact on bovine respiratory disease (BRD) in the field, especially when associated with other pathogens. However, the mechanisms of pathogenesis of IDV in coinfection may be specific to the bacteria, as suggested by the absence of enhanced disease when calves were coinfected with IDV and Mannheimia haemolytica (44). Overall, these findings deepen the knowledge of respiratory coinfections’ pathogenesis and increase the understanding of the molecular mechanisms of mixed airways infections.

## MATERIALS AND METHODS

### Lung donors.

Animal tissues were obtained in accordance to the French regulation on animal experimentation. Three- to six-week old male calves were purchased from the dairy educational farm of the Engineering School for Agriculture of Purpan (INP Toulouse, France), and euthanasia was carried out by intravenous injection of pentobarbital sodium (Dolethal, Vetoquinol) followed by complete exsanguination. The animals displaying respiratory illness or gross lung lesions at necropsy were excluded from the experiments. For each animal, nasal swabs, lung fragments, and sera were collected to assess the pre-exposure to bovine respiratory pathogens. RNA was isolated from nasal swabs and lung fragments using a Qiamp viral RNA mini kit (Qiagen), and the presence of M. haemolytica, Pasteurella multocida, M. bovis, Histophilus somni, bovine coronavirus (BCoV), IDV, BRSV, and BPIV-3 was assessed by real-time PCR using the commercial kit Bio-T respiratory qPCR kits (BioSellal). Serology to detect the presence of anti-IDV antibodies was performed using a hemagglutination inhibition (HI) assay with 0.5% chicken red blood cells derived from specific pathogen-free (SPF) animals (PFIE, INRAE Centre Val de Loire, Nouzilly, France) and 45 min incubation at +4°C (22). Anti-M. bovis antibodies were searched using the commercial ELISA kit ID screen M. bovis Indirect (IDVet). All the lung donors that were positive for one of the above-mentioned pathogens were excluded from the analyses.

### Precision-cut lung slicing and organotypic culture.

The lungs were collected postmortem and they were washed twice with phosphate-buffered saline (PBS) supplemented with 2% of penicillin-streptomycin (PS) (10,000 U/10 mg/mL, Pan Biotech). The PCLSs were obtained from cranial and accessorial lobes. A gelation medium was prepared by melting 2% of low melting point agarose (Thermo Fischer) in RPMI 1640 medium (Thermo Fischer). The gelation medium was cooled and maintained at 42°C and then inflated with a cannula at the bifurcation of the principal bronchi. The lungs were incubated on ice for 30 min to allow agarose polymerization. Biopsy punches of 8 mm in diameter were obtained and sliced using a Krumdieck MD6000 tissue slicer (Alabama Research & Development) in sterile conditions. PCLSs of approximately 100-μm thickness were obtained. After slicing, the PCLSs were placed in P24-well plates containing RPMI medium supplemented with 10% fetal bovine serum (FBS) and 1% PS. Three washing steps with 30 min of incubation at 37°C with 5% CO_2_ were carried out using RPMI medium supplemented with 10% FBS and 1% PS. The PCLSs were then incubated at 37°C with 5% CO_2_ overnight before carrying out the experiments. Two additional washing steps with RPMI medium were done before the infections. After the washing steps, the medium was then replaced with RPMI supplemented with amphotericin B (2.5 μg/mL; Sigma-Aldrich) and ampicillin (0.3 mg/mL; Sigma-Aldrich).

### Viruses, bacteria, and infection of PCLSs.

Influenza D virus isolate D/bovine/France/5920/2014 (29) was propagated on specific pathogen-free embryonated chicken eggs (PFIE, INRAE Centre Val de Loire, Nouzilly, France). IDV was titrated by 50% tissue culture infective dose (TCID_50_) assay using swine testis cells (CRL-1746, ATCC) that were maintained in culture using Dulbecco’s minimum essential medium (DMEM; Gibco) supplemented with 10% heat-inactivated FBS and propagated at 37°C with 5% CO_2_. The cells were seeded in in P96-well plates at a density of 10,000 cells/well, and the following day, the culture medium was removed, the cells were washed once with PBS, and the medium was replaced with DMEM supplemented with 2% PS. Virus stocks were 10-fold diluted in the infection medium and were then inoculated on swine testis cells that were incubated for 5 days at 37°C with 5% CO_2_. The virus titers were revealed by HA assay using 0.5% solution of chicken red blood cells derived from SPF animals (PFIE, INRAE Centre Val de Loire, Nouzilly, France) and 45 min incubation at +4°C. The titers were determined using the Reed-Muench method. M. bovis strain RM16 (45) was grown in SP4 medium at 37°C, and the titer was determined by 10-fold dilutions numeration on SP4 agar plates after 5 days of incubation at 37°C. The PCLSs were washed twice with RPMI medium before the infections. Then, the medium was replaced with RPMI supplemented with amphotericin B (2.5 μg/mL; Sigma-Aldrich) and ampicillin (0.3 mg/mL; Sigma-Aldrich). For replication studies, 10³ TCID_50_ IDV and/or 10³ CFU M. bovis were used (multiplicity of infection [MOI] = 0.001). For innate immunity studies, 10^6^ TCID_50_ IDV and/or 10^6^ CFU M. bovis were used (MOI = 1). Mock allantoic fluid was inoculated in noninfected and M. bovis conditions. To study the effect of a primary IDV infection on M. bovis superinfection, the PCLSs were infected at 0 h with 10^6^ TCID_50_ IDV, and 48 h later, 10^6^ CFU of M. bovis were inoculated in superinfected conditions. The supernatants and lung tissues were then collected at 72 and 96 h p.i.

### Flow cytometry.

The viability of mock lung tissues was assessed by flow cytometry. The PCLSs and their supernatants were briefly spun, the supernatants were discharged, and a solution of 0.25 mg/mL of research-grade Liberase TL (Roche) was added to obtain single-cell suspensions. The PCLSs were then incubated at 37°C for 1 h, and the tissues were dissociated by gentle pipetting. After a washing step using cold PBS and brief centrifugation at 300 × *g* for 5 min, the dissociated cells were stained with 7-amino-actinomycin D (Biolegend) at a final concentration of 5 μg/mL. A final resuspension was done in a final volume of 100 μL of cold PBS supplemented with 2% of FBS, and 50-μL samples were read immediately after on a MACSQuant Analyzer (Miltenyi Biotec). Flow cytometry results were analyzed using FlowJo version 10.7.1 (Tree Star) software.

### LDH activity measure.

To assess the viability of infected and mock PCLSs, 50 μL of supernatants (infected with 10³ TCID_50_ IDV and/or 10³ CFU M. bovis) were collected at different time points and were tested using the Pierce LDH cytotoxicity assay kit (Thermo Scientific), following the manufacturer’s instructions. Positive controls for LDH release were created by incubating PCLSs with a lysis buffer provided by the kit for 1 h at 37°C (“maximum LDH activity”). The optical density of positive controls and PCLS supernatants was read at 490 and 680 nm (background) using a CLARIOStar Plus plate reader (BMG LabTech). The 680-nm absorbance value was subtracted from the 490-nm absorbance before calculating the percentage of cytotoxicity, using the formula [(LDH at 490 nm) − (LDH at 680 nm)] for each sample (“PCLS sample LDH activity”). The percentage of cytotoxicity was then calculated using the following formula: [(PCLS sample LDH activity)/(maximum LDH activity)] × 100.

### RIG-I/MDA-5 and TRL2 stimulation and NF-κB inhibition.

Polyriboinosinic:polyribocytidylic acid (poly[I·C]) (LMW)/LyoVec and Pam3Cys-Ser-(Lys)_4_ (Pam3CSK4) (Invivogen) were selected as agonists to activate specific immune pathways on PCLSs. Poly(I·C) (LMW)/LyoVec is a synthetic double-stranded RNA (dsRNA) polymer that is complexed with a transfection reagent, and it is sensed by cytosolic helicases retinoic acid-inducible gene I and melanoma differentiation-associated gene 5 (RIG-I/MDA-5) in a specific manner (68) and forms known essential signaling pathways upon influenza virus infection (69). Experimental infection in calves with IDV also suggests the activation of this pathway (29). Pam3CSK4 is a synthetic triacylated lipopeptide that is a potent activator of NF-κB pathway upon binding of TLR2/TLR1 receptor and a MyD88-dependent activation, as described during Mycoplasma spp. infection in different species (41–43). To inhibit NF-κB pathway, BAY 11-7082 (Invivogen) was used. BAY 11-7082 was described to inhibit the phosphorylation of IκB-α (which is essential for the release of NF-κB from the cytosolic IκB-α/NF-κB complex) (70), but it was also suggested to inhibit the inflammasome responses indirectly by preventing the nuclear translocation of NF-κB at the priming step but also to directly inhibitory functions on the NLRP3 inflammasome by blocking the sensor’s ATPase activity (71). For stimulations, the PCLSs were treated at 0 h with poly(I·C) at a final concentration of 500 ng/mL, followed by stimulation with Pam3CSK4 at a final concentration of 100 ng/mL 24 h later. To inhibit the NF-κB pathway, the PCLSs were pretreated with BAY 11-7082 at a final concentration of 100 ng/mL, followed by M. bovis infection 6 h later.

### RNA extraction and RT-qPCR.

For pathogen replication, 170 μL of PCLS supernatant was used for RNA extraction using the kit NucleoMag pathogen (Macherey-Nagel) on a KingFisher Flex purification system (Thermo Fisher Scientific). For purification of total RNA from lung tissues, the PCLSs were placed in lysis buffer (provided by the RNA purification kit) in Precellys lysing kit tubes (Bertin Technologies), and tissues were homogenized using a Precellys24 system (Bertin Technologies). The samples were centrifuged at 6,000 × *g* for 5 min, and 300 μL of lysed supernatant was used to extract the total RNA using the NucleoMag RNA kit (Macherey-Nagel) on the same KingFischer Flex system described above. The pathogens were quantified in the supernatant by RT-qPCR (16). M. bovis was quantified using the QuantiNova probe PCR kit (Qiagen) and primers and probe as described (72). RT-qPCR analysis for immune response studies was carried out with RT-qPCR by using the iTaq Universal SYBR green one-step kit (Bio-Rad) using the amplification protocol of the manufacturer. For the relative quantification, we used three previously validated housekeeping genes (HPRT, YWHA7, and GAPDH) to normalize the amount of the target gene (30). The calibration formula 2 − ΔΔ*CT* was used to quantify the relative expression of targeted genes. ΔΔ*CT* represents the following: Δ*CT* (sample) ([*CT*_cytokine gene_ − *CT* geometric mean of the three housekeeping genes] of infected lung cuts) − Δ*CT* (calibrator) ([*CT*_cytokine gene_ − *CT* geometric mean of the three housekeeping genes] of mock lung cuts). For each time point and for each condition, values from infected PCLSs were normalized to the mock PCLS gene expression, and the final results were expressed as fold changes relative to the number of copies of mRNAs in infected PCLSs compared to the mock ones. All the qPCR experiments were performed on a LightCycler 96 real-time PCR system (Roche), and the results were analyzed using the LightCycler 96 Software version 1.1.01320 (Roche). The list of primers used for cytokine amplification in this study is available in Table S1.

### Measurement of IL-1β secretion by ELISA.

To confirm the RT-qPCR results, IL-1β was quantified in mock and infected supernatants using the IL-1β bovine uncoated ELISA kit (Invitrogen), following the manufacturer’s instructions. Prior to quantification, 10 μL of infected and mock supernatants were 10-fold diluted in PBS supplemented with 0.22-μm-filtered bovine serum albumin, and a final volume of 100 μL of diluted samples was used for the test.

### Nitric oxide quantification by Griess assay.

To confirm the RT-qPCR results, the production of nitric oxide was measured in mock and infected supernatants at different time points using the Griess reagent system kit (Promega) and following the manufacturer’s protocol.

### M. bovis strain expressing the red fluorescent protein mCherry.

The M. bovis strain RM16 was transformed with plasmid pOGch expressing the red fluorescent marker mCherry. In pOGch, the mCherry coding sequence was cloned downstream of the gentamicin resistance gene *aacA-aphD* to produce a fusion protein (Gch). The mCherry coding sequence was obtained from Martínez-Torró et al. (73). The primers used for mCherry amplification from the plasmid pCatcherry and the gentamicin marker was amplified with its promoter region from the plasmid pMT85 are available in Table S2 (74). These two regions were assembled by overlap extension PCR using GmF_EcoRI and CherryR_BglII primers. PCRs were performed using the New England Biolabs high-fidelity DNA polymerase. The Gch PCR product was cloned into pGEM-T Easy (Promega) before subcloning at the NotI site of p20-1miniO/T plasmid (75), from which the *tet* gene has been extracted by PstI restriction, to generate pOGch. Plasmid constructions were verified by DNA sequencing. Transformation of pOGch in M. bovis RM16 strain was done as previously described (76). Gentamicin-selected transformants were stored at −80°C. M. bovis mCherry stock cultures were produced in SP4 medium supplemented with gentamicin (50 μg·mL^−1^), and bacterial titers were determined by counting CFU as the wild-type strain.

### Immunofluorescence and confocal microscopy.

For immunofluorescence studies, mock and infected PCLSs were fixed with a 4% paraformaldehyde (PFA) solution for 30 min at room temperature. The PFA was then replaced with a PBS solution and stored at −20°C until the experiments were carried out. PCLSs were permeabilized with 0.5% Triton X-100 in PBS at room temperature under slow agitation for 2 h, followed by 1 h of incubation at room temperature with a blocking buffer (PBS supplemented with 10% horse serum and 0.1% Triton X-100). Primary antibodies were diluted in PBS with 0.1% Triton X-100 and 2% horse serum and incubated overnight at +4°C under slow agitation, followed by five washing steps. The secondary antibodies were diluted in the same buffer used for primary antibodies and were allowed to incubate for 2 h at room temperature under slow agitation protected from direct light. The nuclei were stained with 1 μg/mL of 4′,6-diamidino-2-phenylindole (DAPI) stain (Sigma-Aldrich) for 10 min at room temperature. Propidium iodide (PI) (Bio-Rad) staining was done by using PI at a final concentration of 1 μg/mL. The list of primary and secondary antibodies and their dilutions used is available in Table S3. The PCLSs were mounted on microscopy slides using the ProLong glass hard-set antifade mounting medium (Thermo Fischer), and images were captured with a microscope confocal SP8 STED 3X (Leica). Raw images and z-stacks were first analyzed with Leica application suite X version 3.7.2 (Leica) software. For z-stacks, the confocal plans of interest were selected for each channel, and maximum intensity projections were created, using Fiji ImageJ (77), to obtain two-dimensional images. The brightness and contrast were adjusted for each channel individually compared to control conditions. The red, green, and cyan channels were then merged together to obtain the final images.

### Electron microscopy.

Noninfected and infected PCLSs (infected 10^6^ TCID_50_ IDV and/or 10^6^ CFU M. bovis) were fixed at different time points with 2% glutaraldehyde (EMS) (CliniSciences) in 0.1 M sodium cacodylate (pH 7.4) buffer (CliniSciences) at room temperature. After washing three times in 0.2 M sodium cacodylate buffer, PCLSs were postfixed with 1% aqueous osmium tetroxide (Electron Microscopy Sciences) for 1 h at room temperature, dehydrated in a graded series of ethanol at room temperature, and embedded in Epon (Sigma-Aldrich). After polymerization, ultrathin sections (100 nm) were cut on a UC7 (Leica) ultramicrotome and collected on 200-mesh grids. Sections were stained with uranyl acetate and lead citrate before observations on a Jeol 1400JEM (Tokyo, Japan) transmission electron microscope equipped with an Orius 1000 camera and digital micrograph.

### Statistical analysis.

All of the experiments were performed with three biological replicates for each condition. Two-way analysis of variance (ANOVA) with Tukey’s multiple comparisons method was performed on GraphPad Prism version 9.3.1 (GraphPad Software, San Diego, California, http://www.graphpad.com). The represented *P* values in figures are as follows: *, *P* < 0.05; **, *P* < 0.01; ***, *P* < 0.001; and ****, *P* < 0.0001. The data are expressed as arithmetic mean values ± standard error of the mean (SEM).
