# Supplementary material for: The Activation of the RIG-I/MDA5 Signaling Pathway upon Influenza D Virus Infection Impairs the Pulmonary Proinflammatory Response Triggered by Mycoplasma bovis Superinfection
Source: J Virol. 2023 Jan 24;97(2):e01423-22. doi: 10.1128/jvi.01423-22 (PMC9972951; doi:10.1128/jvi.01423-22)
Supplement: Supplemental file 1 — Fig. S1 to S5 and Tables S1 to S3. Download jvi.01423-22-s0001.pdf, PDF file, 1.3 MB [file jvi.01423-22-s0001.pdf]

Figure S1. LDH activity measure in infected PCLS

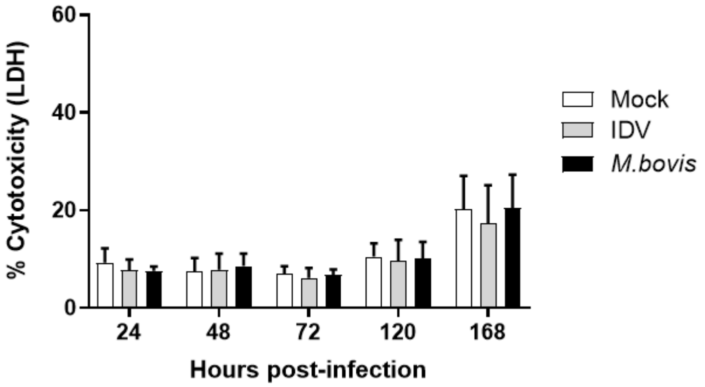

Figure S2. Confocal microscopy studies of IDV and M. bovis distribution on PCLS. In all the images, nuclei were stained with DAPI (cyan). A) and B) IDV (red) infecting Club cells (green) in bronchioles at 48 hours p.i. C) IDV (red) infecting endothelial cells (green) at 48 hours p.i. D) Z-stack image of IDV (green) infecting bronchial cells at 48 hours p.i. E) and F) IDV (green) infecting a cell expressing MHC-II (red) at 120 hours p.i. G) *M. bovis* mCherry (red) infecting Club cells (green) at 120 hours p.i. H) *M. bovis* mCherry (red) infecting an alveolar macrophage (green) at 72 hours p.i.

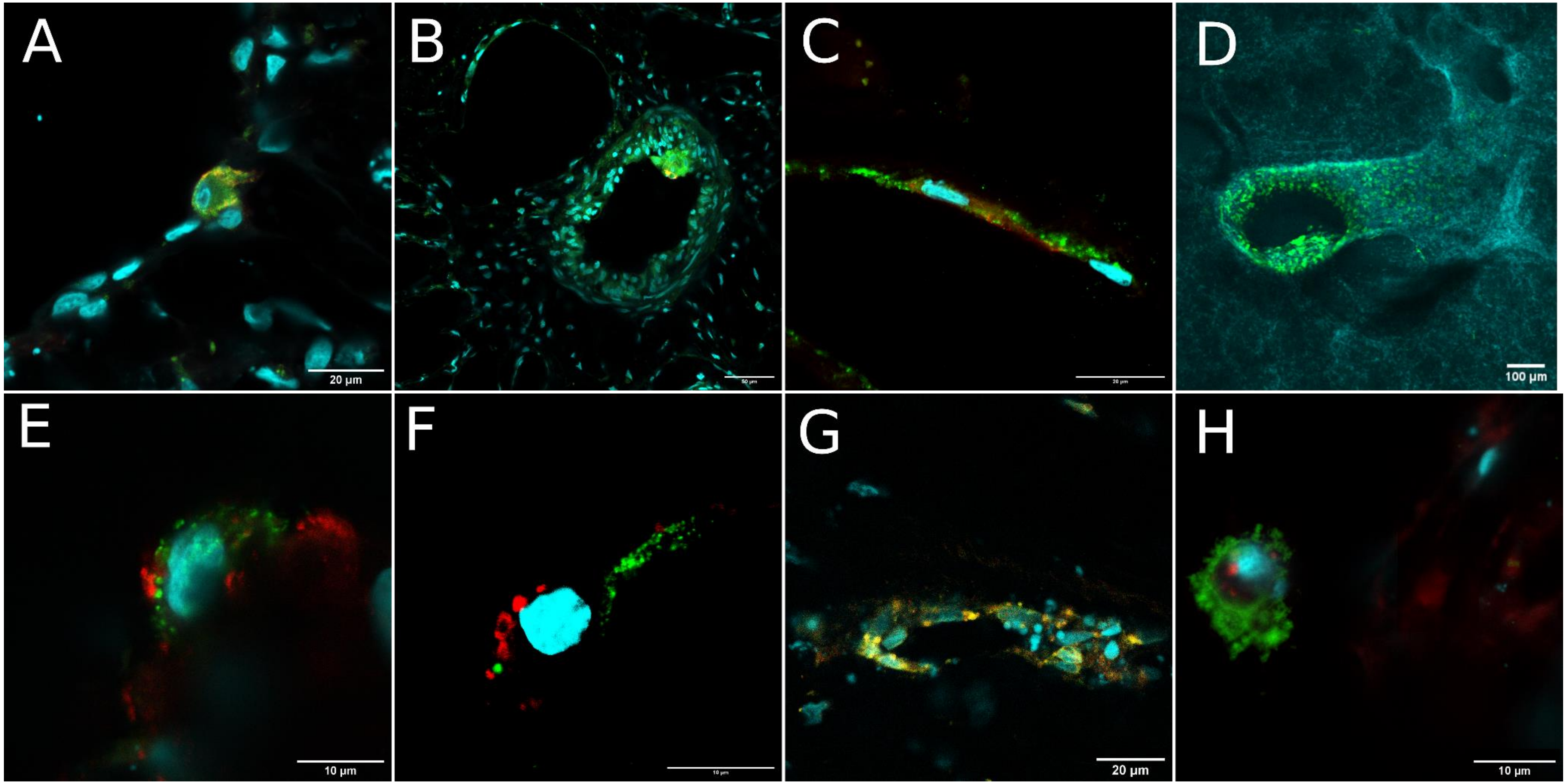
A
20 µm
B
C
D
100 µm
E
10 µm
F
G
20 µm
H
10 µm

Figure S3. Confocal images of mock PCLS at 48 hours p.i.

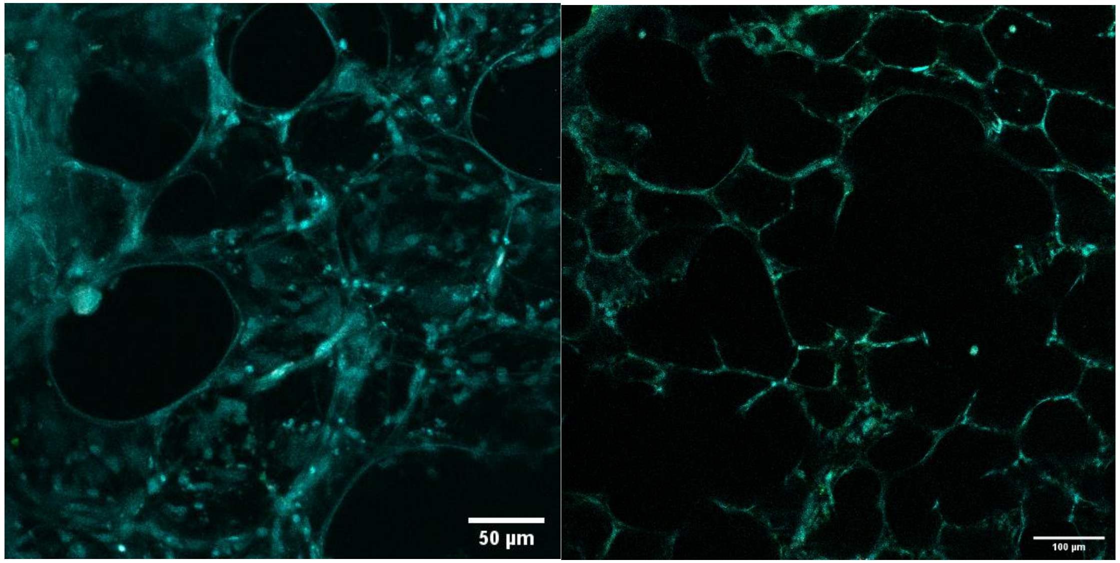

Figure S4. Transmission electronic microscopy image on co-infected PCLS showing a phagophore structure (double-membrane vesicle) at 24 hours p.i. The PCLS were infected with an MOI of 1 (10^6 TCID50/PCLS for IDV, 10^6 CFU/PCLS for *M. bovis*).

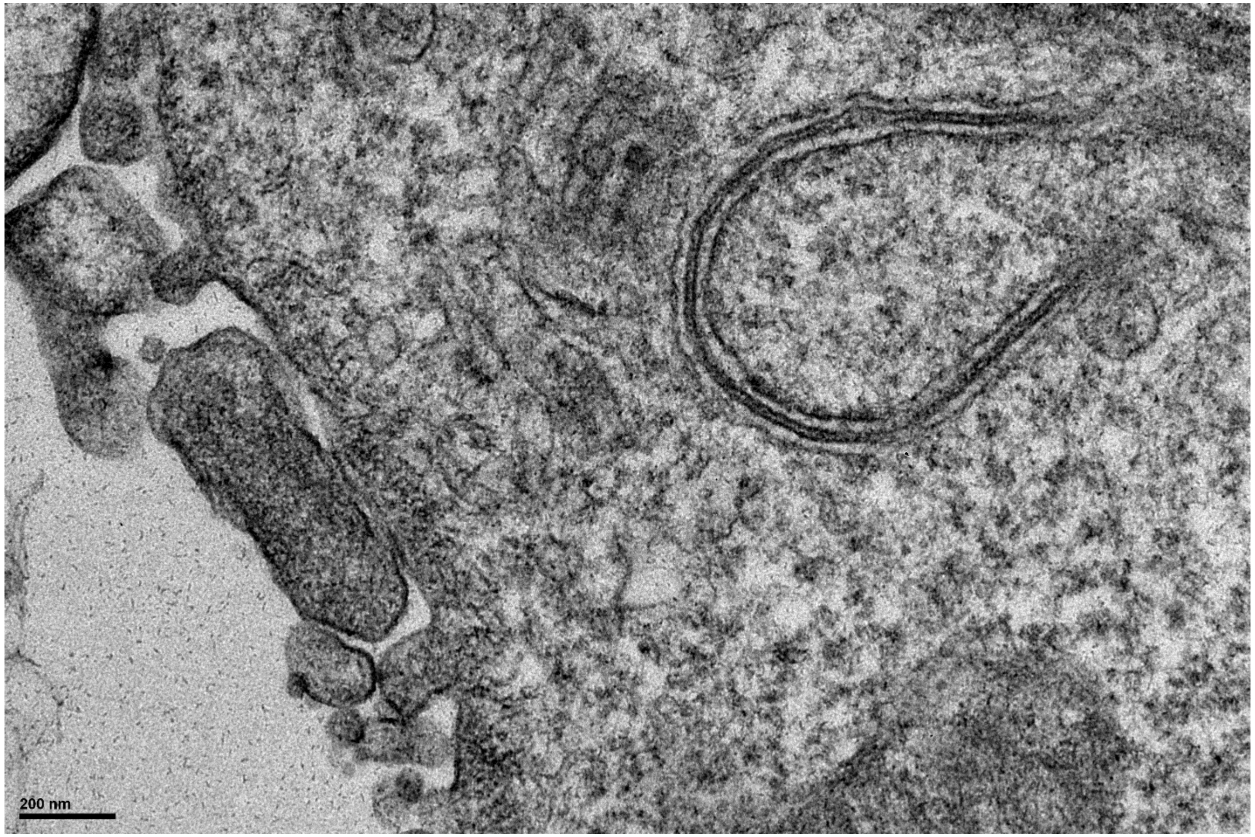
200 nm

Figure S5. Impact of IDV on the pro-inflammatory and antibacterial immune response against M. bovis at different time points. A) Transcriptomic analysis of the expression of different cytokines measured by RT-qPCR in infected lung tissue. The dotted line represents the fold change of mock PCLS. The PCLS were infected with an MOI of 1 (10^6 TCID50/PCLS for IDV, 10^6 CFU/PCLS for M. bovis). The plotted values above bars represent the mean for each group.

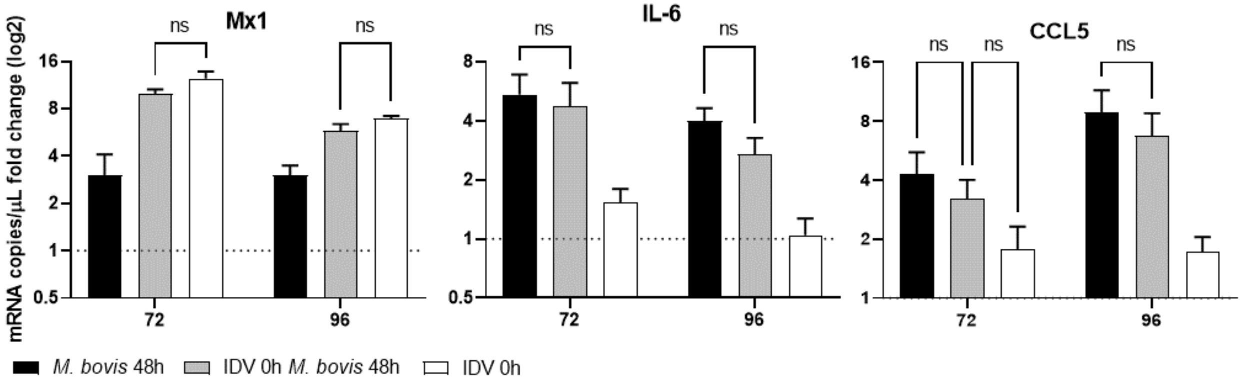

Table S1. Primers used for RT-qPCR experiments

Target	Reference
YWHA7	[1]
GAPDH	[2]
HPRT	[1]
IL-8 (CXCL8)	[1]
Mx1	[1]
ISG15	[1]
IL-10	[1]
IFN-γ	[1]
IFN-β	[1]
IL-6	[1]
CCL5	[1]
TNF-α	[1]
IL-1β	[1]

IP-10 (CXCL10)	[3]
iNOS (NOS2)	[4]
IL-17A	[5]

Table S2. Primers used for the construction of *M. bovis* fluorescent strain

mCherry amplification from plasmid pCatcherry	GmvsCh_F	5’-ATTTATAAAAGGACTTATAAAGATagcaagggcgaggaggataacat-3’
	CherryR_BglII	5’-TTCAGATCTGGATCCTTACTTGTACAGGTCGTC-3’
Gentamicin marker amplification from plasmid pMT85	GmF EcoRI	5’-GATCTGAATTCGCATTTTACACAGGAGTCTGGA-3’
	GmvsCh R	5’-ATGTTATCCTCCTCGCCCTTGCTATCTTTATAAGTCCTTTTATAAAT-3’

Table S3. Antibodies and stainings used for immunofluorescence

Target cells	Primary antibody (or staining)	Dilution/ concentration	Secondary antibody	Dilution
IDV	Polyclonal serum anti-IDV nucleoprotein (Rabbit) [6]	1:200	Donkey anti-rabbit 488 (711-546-152, Jacskon Research)	1:2000
IDV	Monoclonal 3G3 [Mouse hybridoma against influenza D virus HE] [7]	1:200	Goat anti-Mouse IgG (H+L) Highly Cross-Adsorbed Secondary Antibody, Alexa Fluor™ 594 (A-11032 Invitrogen)	1:1000
Club cells	Anti-Clara Cell Secretory Protein Antibody (Rabbit) (Sigma-Aldrich)	1:200	Donkey α rabbit 488 (711-546-152, Jacskon Research)	1:1000

Alveolar macrophages, T cells	MHC Class II DQ antibody | CC158 (Biorad)	1:500	Goat anti-Mouse IgG (H+L) Highly Cross-Adsorbed Secondary Antibody, Alexa Fluor™ 594 (A-11032 Invitrogen), Goat anti-Mouse IgG2a Cross-Adsorbed Secondary Antibody, Alexa Fluor™ 488 (A-21131 Invitrogen)	1:1000
Endothelial cells	Anti-Von Willebrand Factor antibody (Rabbit) (ab6994, abcam)	1:200	Goat anti-Rabbit IgG (H+L) Secondary Antibody, FITC (65-6111 Invitrogen)	1:1000
Necrotizing cells	Propidium Iodide (Biorad)	1µg/mL		
Nuclei	DAPI (Sigma-Aldrich)	0,5 µg/mL		
β-tubulin	Monoclonal Anti-β-Tubulin−Cy3 antibody produced in mouse (C4585-.2ML) (Sigma-Aldrich)	1:500		

References

1. Lion A, Secula A, Rançon C, Boulesteix O, Pinard A, Deslis A, et al. Enhanced Pathogenesis Caused by Influenza D Virus and Mycoplasma bovis Coinfection in Calves: a Disease Severity Linked with Overexpression of IFN-γ as a Key Player of the Enhanced Innate Immune Response in Lungs. Kibenge FSB, editor. Microbiol Spectr. 2021;9. doi:10.1128/SPECTRUM.01690-21

2. Gonzalez DD, Rimondi A, Perez Aguirreburualde MS, Mozgovoj M, Bellido D, Wigdorovitz A, et al. Quantitation of cytokine gene expression by real time PCR in bovine milk and colostrum cells from cows immunized with a bovine rotavirus VP6 experimental vaccine. Res Vet Sci. 2013;95: 703–708. doi:10.1016/J.RVSC.2013.03.016

3. Sakumoto R, Iga K, Hayashi KG, Fujii S, Kanahara H, Hosoe M, et al. Gene expression of CCL8 and CXCL10 in peripheral blood leukocytes during early pregnancy in cows. J Anim Sci Biotechnol. 2018;9: 1–11. doi:10.1186/S40104-018-0263-Z/FIGURES/6

4. Jensen K, Stevens JM, Glass EJ. Interleukin 10 knock-down in bovine monocyte-derived macrophages has distinct effects during infection with two divergent strains of Mycobacterium bovis. PLoS One. 2019;14: e0222437. doi:10.1371/JOURNAL.PONE.0222437

5. Cunha P, Vern Y Le, Gitton C, Germon P, Foucras G, Rainard P. Expansion, isolation and first characterization of bovine Th17 lymphocytes. Sci Reports 2019 91. 2019;9: 1–14. doi:10.1038/s41598-019-52562-2

6. Donchet A, Oliva J, Labaronne A, Tengo L, Miloudi M, C.A. Gerard F, et al. The structure of the nucleoprotein of Influenza D shows that all

Orthomyxoviridae nucleoproteins have a similar NPCORE, with or without a NPTAIL for nuclear transport. Sci Reports 2019 91. 2019;9: 1–14. doi:10.1038/s41598-018-37306-y

7. Moreno A, Lelli D, Lavazza A, Sozzi E, Zanni I, Chiapponi C, et al. MAb-based competitive ELISA for the detection of antibodies against influenza D virus. 2019;66: 268–276. Available: https://pubmed.ncbi.nlm.nih.gov/30179314/
